# The Prevalence, Severity, and Predictive Factors of Restless Legs Syndrome in Pregnancy

**DOI:** 10.7759/cureus.44884

**Published:** 2023-09-08

**Authors:** Hasan Turan, Suna Aşkın Turan, Zafer Butun, Masum Kayapınar

**Affiliations:** 1 Obstetrics and Gynecology, University of Health Sciences, Mersin City Hospital, Mersin, TUR; 2 Neurology/Pain Management, University of Health Sciences, Mersin City Hospital, Mersin, TUR; 3 Obstetrics and Gynecology/Maternal Fetal Medicine, University of Health Sciences, Eskişehir City Hospital, Eskişehir, TUR; 4 Obstetrics and Gynecology/Maternal Fetal Medicine, University of Health Sciences, Mersin City Hospital, Mersin, TUR

**Keywords:** restless legs syndrome, thyroid-stimulating hormone, gravida, serum vitamin b12, age, hereditary, ferritin, familial, pregnancy, rls

## Abstract

Objectives: The current study aimed to search the prevalence and severity of restless legs syndrome (RLS) in pregnancy according to the three trimesters and predictive factors of RLS in pregnancy based on validated diagnostic tools and a thorough literature review.

Methods: The cross-sectional descriptive study included 500 pregnant women without comorbidities who were interviewed face-to-face. Age, height, weight, week of pregnancy, smoking, alcohol, caffeine use, regular exercise, and lab test results from the last visit were all included in the data. Only women satisfying the RLS diagnostic criteria were given the Restless Legs Syndrome Rating Scale.

Results: The prevalence of RLS was found to be 29.2% with the highest rate in the third trimester (64.4%). In all trimesters, low ferritin (first trimester: p = 0.004; second trimester: p < 0.001; third trimester: p < 0.001), folic acid (first trimester: p = 0.001; second trimester: p < 0.001; third trimester: p < 0.001), vitamin B12 (first trimester: p = 0.003; second trimester: p < 0.001; third trimester: p < 0.001), and hemoglobin (first trimester: p < 0.001; second trimester: p < 0.001; third trimester: p < 0.001) levels were associated with RLS. In the second and third trimesters, low magnesium (p < 0.001 and p < 0.001, respectively) and high creatinine (p = 0.027 and p < 0.001, respectively) levels were associated with RLS. Higher thyroid-stimulating hormone and free T4 levels were associated with RLS in the third trimester but not in the first and second trimesters (median: 2.4 vs. 2.1, p < 0.001; median: 1.5 vs. 1.2, p < 0.001). In the multivariate regression analysis, age (p = 0.034, OR: 1.060, 95% CI: 1.005-1.119), present BMI (p < 0.001, OR: 1.8884, 95% CI: 1.597-2.222), BMI before conception (p < 0.001, OR: 0.607, 95% CI: 0.513-0.718), gravida (p < 0.001, OR: 2.172, 95% CI: 1.547-3.049), low ferritin level (p < 0.001, OR: 6.396, 95% CI: 0.00744-0.010405), low vitamin B12 (p < 0.001, OR: 10.347, 95% CI: 0.00120-0.00176), low folate (p < 0.001, OR: 5.841, 95% CI: 0.00616-0.01240), RLS history before conception (p = 0.013, OR: 4.963, 95% CI: 1.402-17.57), and RLS family history (p < 0.001, OR: 7.914, 95% CI: 0.18760-0.31151) were found to be predictive factors for RLS in pregnancy.

Conclusion: More attention is needed to RLS during pregnancy to prevent or treat this syndrome.

## Introduction

Restless legs syndrome (RLS) is a sensorimotor disorder characterized by an urgent need to move the legs in response to a feeling of discomfort in the lower extremities. Symptoms typically manifest during inactivity and disappear with activity [1]. The international RLS diagnostic criteria were established in 1995, revised by the American National Institute of Health (NIH) in 2003, and updated by the International Restless Legs Syndrome Study Group (IRLSSG) in 2014 [2]. RLS is categorized as idiopathic or symptomatic depending on the existence of coexisting diseases such as renal insufficiency, peripheral neuropathy, anemia, or pregnancy [3].

Recent systematic reviews assessed that RLS prevalence during pregnancy ranges from 11% to 34% [4-12], typically two to three times higher than in nonpregnant women in general. According to Turkish studies, the prevalence of RLS during pregnancy ranges from 19% to 26% [5-9,11,12]. RLS during pregnancy is linked to gestational hypertension, preeclampsia, poor sleep and quality of life, daytime lethargy, and depression. History of RLS before conception, RLS during a previous pregnancy, coffee consumption before conception, peptic ulcer disease, hemoglobin < 11 g/dL, and inadequate supplementation of iron and folate during pregnancy, especially when iron deficiency is present, have been identified as risk factors for the development of RLS during pregnancy [13-29].

There is literature from Turkey about the prevalence and risk factors for RLS during pregnancy. However, a few studies have used the RLS severity scale and diagnostic criteria. In addition, a few epidemiological and clinical data have been analyzed to determine the risk factors of RLS. Little research has been done on the link between RLS and biochemical markers such as hemoglobin, thyroid hormones, folate, and vitamin B12 in pregnant women across all trimesters [6,27,28]. The current study aims to investigate the prevalence and severity of RLS in pregnancy according to the three trimesters and the predicting factors of RLS in pregnancy, using validated diagnostic instruments and a thorough review of the literature. The study's secondary objective was to assess if the relationship between RLS and biochemical markers varied by trimester.

## Materials and methods

The current descriptive cross-sectional study wanted to determine the prevalence and predictive factors of RLS during pregnancy in all trimesters. The study was approved by the local ethics committee (ESH/GOEK 2022/17) and written informed consent was obtained from each participant. A neurologist was consulted for the pregnant woman who had been diagnosed with RLS. The study complied with the Declaration of Helsinki's ethical standards and was reported following the cross-sectional STROBE (Strengthening the Reporting of Observational Studies in Epidemiology) checklist.

Population and sample size of the study

**Figure 1:**
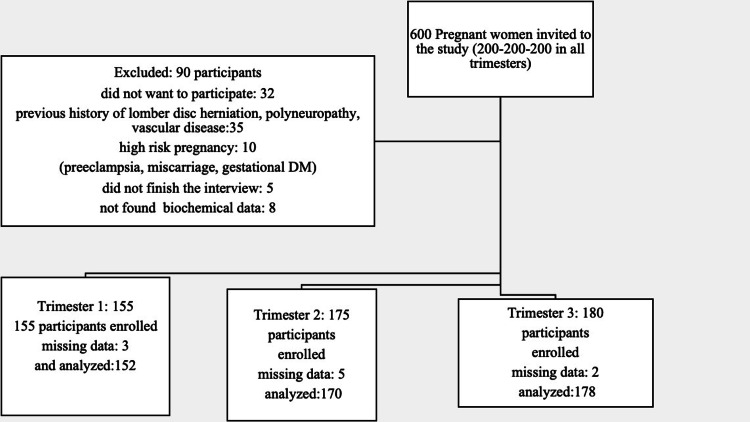
Flowchart diagram of the study DM: diabetes mellitus.

The 5000 pregnant women who applied for the gynecology and obstetrics outpatient clinics at the Health Science University Eskişehir and Mersin City Training and Research Hospitals between January 3, 2023, and June 6, 2023, made up the cross-sectional prospective descriptive study population universe. Using a power analysis to calculate the sample size, the prevalence of RLS was estimated to be between 19% and 26% (sample error: 0.01; power: 95%) [7,8]. The minimal number of participants in the study sample was determined to be 280. In the trial, 500 pregnant women in all three trimesters were randomly selected from the two clinics and interviewed once (Figure 1).

Pregnant women aged 18-35 years with no comorbidities such as diabetes, kidney disease, thyroid disease, radiculopathy, plexopathy, polyneuropathy, peripheral nerve lesions, vascular diseases, psychiatric disease, parkinsonism or other movement disorders, other sleep disorders (narcolepsy, rapid eye movement disorder, or obstructive sleep apnea syndrome), or no high-risk pregnancies (preeclampsia, gestational diabetes mellitus) were enrolled.

Procedure

Pregnant women were given the data collection tools during an in-person interview with the researchers. On the basis of a review of the relevant literature, the authors created a questionnaire to capture the data. The form consisted of three sections: epidemiological and clinical data, RLS diagnostic criteria, and the Restless Legs Syndrome Rating Scale (RLSRS). Age, height, weight before pregnancy, current weight, pregnancy week, smoking/alcohol/caffeine status, regular exercise, and laboratory parameters, including folic acid, vitamin B12, ferritin, and thyroid function tests, were recorded as epidemiological and clinical data. Only women who met the diagnostic criteria for RLS were given the RLSRS. Interviews were conducted at random once per week for three months.

IRLSSG Diagnostic Criteria for RLS

This questionnaire was developed in 1995 based on the experiences of patients and includes five questions. Patients who respond “yes” to all five questions are diagnosed with RLS [7]. Patients were also analyzed by the neuroalgologist author.

The Restless Legs Syndrome Rating Scale

The RLSRS was developed by IRLSSG [9]. It includes 10 items, which are scored from none (score 0) to very severe (score 4). The minimum and maximum scores obtained on the scale are 0 and 40, respectively. The severity is classified as mild (score 0-10), moderate (score 11-20), severe (score 21-30), and very severe (score 31-40). The Turkish version of the scale has been used in many studies in Turkey [5-9,11,12].

Statistical analyses

IBM SPSS Statistics for Windows, version 22.0 software (IBM Corp., Armonk, NY) was used to perform the statistical analysis. Mean, SD, median, minimum, maximum, frequency, and percentage values were used as descriptive statistics. The distribution of the variables was evaluated using the Kolmogorov-Smirnov test. Student’s t-test was used for continuous variables and a chi-square test for categorical variables. Pearson correlation analysis was applied to examine relationships between variables. Multivariate linear regression analysis was employed to determine predictive factors for RLS during pregnancy. The results were evaluated with a confidence interval of 95% and a significance level of p < 0.05.

## Results

This study included 500 pregnant women including all three trimesters. Demographic and clinical characteristics of the study participants with and without RLS are provided in Table 1.

**Table 1:** Sociodemographic features of the participants *: statistically significant; RLS: restless legs syndrome.

	RLS (+)	RLS (-)	P
First trimester (n, %)	7, 4.8%	145, 41%	Between trimester 1 and trimester 3 (p < 0.001)
Second trimester (n, %)	45, 30.8%	125, 35.3%	
Third trimester (n, %)	94, 64.4%	84, 23.7%	
Total (n, %)	146, 29.2%	354, 71.8%	
Age (mean, SD)	29.69( 5.32)	27.08 (5.34)	<0,001*
BMI (present), mean (SD)	29.12 (4.63)	24.43 (4.35)	<0.001*
BMI (before pregnancy, mean (SD)	25.45 (4.43)	22.82 (3.99)	<0.001*
Smoking quit before pregnancy	11 (7.5%)	12 (3.4%)	0.038*
Alcohol quit before pregnancy	6 (4%)	4 (1%)	0.0071*
Coffee/tea more than 1 cup/a day	96 (65.8%)	187 (52.8%)	0.011*
Gravida			
1	24 (16.4%)	189 (53.9%)	<0.001*
2	63 (43.2%)	107 (30.2%)	
3	51 (34.9%)	50 (14.1%)	
4	8 (5.5%)	8 (2.3%)	
0	124 (84.9)	23 (91.2%)	
Abortus	18 (12.3%)	9 (8.2%)	0.033*
RLS before pregnancy	15 (10%)	6 (2%)	<0.001*
RLS family history	101 (69%)	75 (21%)	<0.001*
Previous preeclampsia	12 (8%)	13 (4%)	<0.034*
Exercise regularly, yes	116 (80%)	316 (88%)	<0.001*
Oral ferritin use regularly	128 (88%)	327 (92%)	0.098
Magnesium use	63 (43%)	89 (25%)	<0.001*
Folic acid use	63 (43%)	162 (46%)	0.593
Vitamin supplement use	118 (81%)	318 (90%)	0.006*

**Figure 2:**
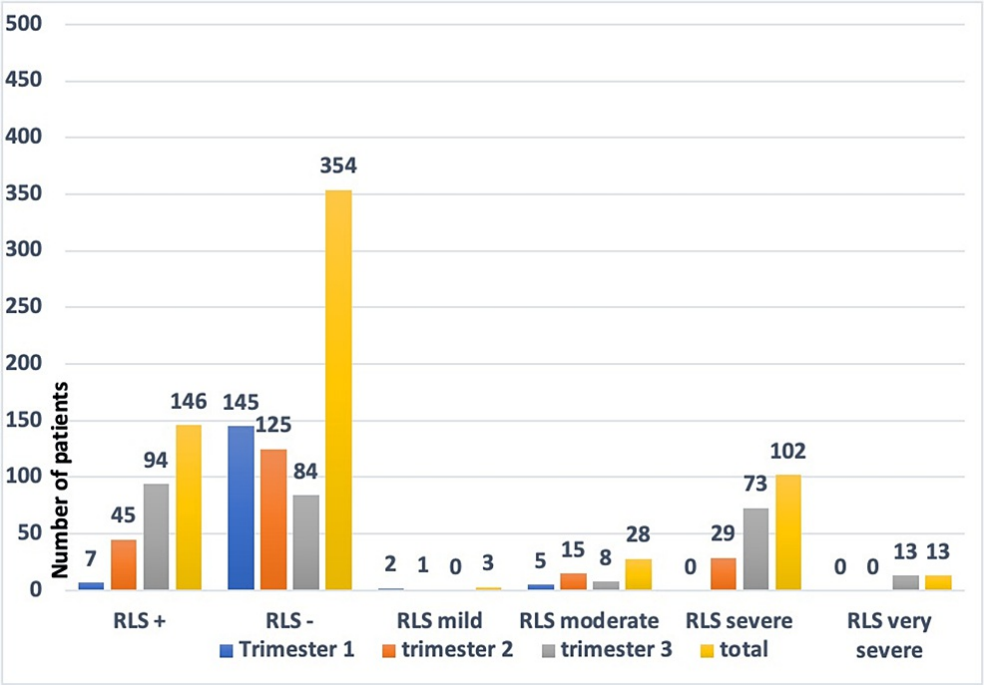
RLS prevalence and severity according to trimesters RLS: restless legs syndrome.

The percentage of women who fulfilled the criteria of RLS diagnosis was 29.2% (n = 146/500) in total, with 64.4% in the third trimester, 30.8% in the second trimester, and 4.8% in the first trimester. The severity of RLS was higher in the third trimester (p < 0.001) (Figure 2).

The mean age in the RLS group was higher than the non-RLS group (29.69 + 5.32 and 27.08 + 5.34; p < 0.001). The body mass index (BMI) at present and before conception were both higher in the RLS group than the non-RLS group (29.12, p < 0.001; 25.45, p < 0.001, respectively). Smoking and alcohol consumption before conception were also associated with the RLS group (p = 0.038 and p = 0.0071, respectively). More than one cup of coffee/tea consumption during pregnancy was higher in the RLS group (65.8%, p = 0.011). Gravida, previous preeclampsia, and migraine were associated with RLS (p < 0.001, p < 0.034, and p = 0.013, respectively). Exercise and vitamin supplement use were higher in the non-RLS group (p < 0.001 and p = 0.006, respectively). RLS history in the family and RLS before conception were associated with RLS (p < 0.001 and p < 0.001, respectively) (Table 1).

The biochemical parameters are detailed in Table 2 according to trimesters. In all trimesters, low ferritin (first trimester: p = 0.004; second trimester: p < 0.001; third trimester: p < 0.001), folic acid (first trimester: p = 0.001; second trimester: p < 0.001; third trimester: p < 0.001), vitamin B12 (first trimester: p = 0.003; second trimester: p < 0.001; third trimester: p < 0.001), and hemoglobin (first trimester: p < 0.001; second trimester: p < 0.001; third trimester: p < 0.001) levels were associated with RLS. In the second and third trimesters, low magnesium (p < 0.001 and p < 0.001, respectively) and high creatinine (p = 0.027 and p < 0.001, respectively) levels were associated with RLS. Higher thyroid-stimulating hormone and free T4 levels were associated with RLS in the third trimester but not in the first and second trimesters (median: 2.4 vs. 2.1, p < 0.001; median: 1.5 vs. 1.2, p < 0.001).

**Table 2:** Biochemical parameters of the participants according to all trimesters *: statistically significant; RLS: restless legs syndrome; TSH: thyroid-stimulating hormone; T4: thyroxine.

	Trimester 1			Trimester 2			Trimester 3		
	RLS (+), Median (Q1-Q3)	RLS (-), Median (Q1-Q3)	P	RLS (+), Median (Q1-Q3)	RLS (-), Median (Q1-Q3)	P	RLS (+), Median (Q1-Q3)	RLS (-), Median (Q1-Q3)	P
Hemoglobin	11 (10-12)	12 (11-16)	<0.001	10 (9-12)	11 (10-13)	<0.001	10 (8-12)	11 (9-13)	<0.001
Ferritin	15 (12-18)	32 (24-40)	0.004*	10 (9-12)	18 (15-20)	<0.001*	10 (8-11)	15 (12-20)	<0.001*
Magnesium	1.9 (1.8-2.1)	1.9 (1.8-2.1)	0.751	1.8 (1.7-1.9)	1.9 (1.89-2.1)	<0.001*	1.8 (1.6-1.9)	1.9 (1.8-2.2)	<0.001*
Urea	28 (24-32)	30 (25-34)	0.979	30 (26-34)	30 (25-37)	0.582	30 (25-38)	30 (25-35)	0.205
Creatinine	0.8 (0.8-0.9)	0.8 (0.7-0.9)	0.687	0.9 (0.8-1)	0.8 (0.8-0.9)	0.027*	0.95 (0.9-1.1)	0.8 (0.8-0.9)	<0.001*
Aspartate transaminase	20 (18-20)	20 (18-20)	0.799	20 (18-21)	20 (18-21)	0.552	20 (19-22)	20 (18-20)	0.148
Alanine transaminase	19 (18-20)	20 (18-20)	0.379	20 (18-22)	20 (18-20)	0.71	20 (18-22)	20 (18-20)	0.034*
TSH	2.5 (2.2-2.7)	2.4 (2.1-2.6)	0.428	2.4 (2.2-2.5)	2.4 (2.2-2.5)	0.262	2.4 (2.2-2.8)	2.1 (1.9-2.45)	<0.001*
Free T4	1.3 (0.8-1.6)	1.4 (1.1-1.8)	0.289	1.2 (1.1-1.7)	1.5 (1.1-1.8)	0.123	1.5 (1.1-1.8)	1.2 (0.9-1.55)	0.001*
Vitamin B12	180 (140-200)	240 (198-340)	0.003*	180 (150-190)	258 (198-350)	<0.001*	175 (140-198)	269 (227-332.5)	<0.001*
Folic acid	8 (2-9)	20 (14-25)	<0.001*	8 (6-10)	12 (8-19)	<0.001*	8 (5-10)	18 (10-22.5)	<0.001*

In the multivariate regression analysis (Table 3), age (p = 0.034, OR: 1.060, 95% CI: 1.005-1.119), present BMI (p < 0.001, OR: 1.8884, 95% CI: 1.597-2.222), BMI before conception (p < 0.001, OR: 0.607, 95% CI: 0.513-0.718), gravida (p < 0.001, OR: 2.172, 95% CI: 1.547-3.049), low ferritin level (p < 0.001, OR: 6.396, 95% CI: 0.00744-0.010405), low vitamin B12 (p < 0.001, OR: 10.347, 95% CI: 0.00120-0.00176), low folate (p < 0.001, OR: 5.841, 95% CI: 0.00616-0.01240), RLS history before conception (p = 0.013, OR: 4.963, 95% CI: 1.402-17.57), and RLS family history (p < 0.001, OR: 7.914, 95% CI: 0.18760-0.31151, respectively) were found to be predictor factors for RLS in pregnancy.

**Table 3:** Multivariate regression analysis of predictive factors of RLS in pregnancy *: statistically significant; RLS: restless legs syndrome.

Risk factors	P-value	Odds ratio	95% confidence interval
Age	0.034*	1.060	1.005-1.119
BMI present	<0.001*	1.8884	1.597-2.222
BMI before pregnancy	<0.001*	0.607	0.513-0.718
Comorbidity	0.056	4.986	0.962-25.829
Preeclampsia	0.489	1.497	0.477-4.698
RLS history before pregnancy	0.013*	4.963	1.402-17.57
RLS history in the family	<0.001*	7.914	0.18760-0.31151
Magnesium use	0.904	1.037	0.572-1.882
Gravida	<0.001*	2.172	1.547-3.049
Ferritin	<0.001*	6.396	0.0744-0.01405
Magnesium	0.366	-0.905	-0.09141-0.03376
Vitamin B12	<0.001*	10.347	0.00120-0.00176
Folate	<0.001*	5.841	0.00616-0.01240

## Discussion

In the current cross-sectional study, we demonstrated that 29.2% of pregnant women had RLS as diagnosed by standard IRLSSG criteria, and more than half of these women were in the third trimester and had severe RLS symptoms. Age, BMI, gravida, parity, use of alcohol and tobacco prior to conception, coffee use, prior RLS history, and familial RLS history were all associated with RLS. In each trimester, RLS was linked to low levels of ferritin, hemoglobin, folic acid, and vitamin B12. In the second and third trimesters, RLS was correlated with low magnesium and elevated creatinine levels. In the third trimester but not in the first or second, higher levels of free T4 and thyroid-stimulating hormone were linked to RLS. Age, BMI, familial RLS history, RLS history before conception, gravida, low ferritin, vitamin B12, and folate levels were identified to be predictors of RLS during pregnancy in the regression analysis.

RLS prevalence during pregnancy in different countries has been reported at a range of 13.5-34%, in line with our findings [1-19]. According to Turkish studies, the prevalence of RLS during pregnancy ranges from 15.4% to 61.2% [5-9,11,12]. The reason for the wide range might be a variety of the exclusion and inclusion criteria of the studies. In the current study, we excluded the comorbidities that can mimic or cause RLS. One of the two pregnant women in the third trimester was found to have RLS in the current study, but in the previous studies, the ratio was 1/3. Also, the ratio of RLS in the first trimester was found very low in our study compared with the literature. When assessing RLS severity, the majority of the participants (102/146) with RLS had severe symptoms, especially in the second and third trimesters. This ratio was a bit higher than the literature from Europe but consistent with the data from Turkey and developing countries [6,13,19,25,26]. The differences might be related to population diversity.

Previous data on the evidence of a link between age and RLS are contradictory. Some authors discovered the association [13,15,17], while others did not [4,19]. Our study discovered pregnancy age as a risk factor for RLS. We did not use a cut-off value for age. As the age increases, so does the prevalence of RLS. In the scientific literature, gestational diabetes and preeclampsia are established as risk factors. In the present study, high-risk pregnancies were excluded; yet, previous preeclampsia was identified as a risk factor for RLS, but the regression model did not reveal a statistically significant relationship. Future well-designed investigations investigating the link between RLS and preeclampsia are required. Prior to conception and during pregnancy, BMI was found to be a risk factor in the current study, consistent with previous research [6,13,15,19].

Iron deficiency is a well-known cause of RLS during pregnancy. Numerous studies have shown that iron plays a role in the etiopathogenesis of RLS [19,27]. Iron, ferritin, and serum folate levels drop during pregnancy, as does hemoglobin, another iron indication [1,28]. There is evidence that the number of pregnancies can affect iron levels. If not restored between pregnancies, it tends to decline with each subsequent pregnancy. Thus, multiparity appears to be associated with a higher risk of developing RLS [15]. In the present study, ferritin, folate levels, and multiparity were identified as predictors in each trimester.

Several studies have demonstrated that RLS during pregnancy may be caused by genetic factors [15,16,20,22]. Familial RLS is substantially more common in pregnant women with this condition than in women with secondary manifestations or without it [22]. RLS in prior pregnancies or RLS in the past when not pregnant have also been identified as risk factors in the literature [3,4,6,13]. In our study, the risk increased ninefold in women with Familial RLS or fivefold in women with RLS history. Further genetic investigations are needed in women with pregnancy-related RLS to better understand the link.

Vitamin B12 was discovered to be a risk factor in this study, increasing the risk ten-fold. Geng et al. identified an independent association between decreased serum vitamin B12 levels and the development of RLS. The authors hypothesized that vitamin B12 may be related to dopamine dysfunction mechanisms in RLS [29]. But more research is needed to see the relation.

This cross-sectional study was conducted in two significant metropolises in Turkey, with participants in each of the three trimesters. A neurologist conducted the interview using validated tools. The findings may help health professionals and future research.

Our research also has some limitations. Despite the fact that the current study had more participants than the sample size, the data do not reflect all pregnant women in Turkey. Despite the fact that the study population was large enough to assess RLS prevalence, it was too small to uncover specific predictors of RLS during pregnancy. Second, no prospective follow-up was conducted to determine if RLS recurred or persisted in subsequent trimesters or after delivery. Although validated and widely used, the diagnosis of RLS was dependent on a self-report questionnaire (IRLSS). Third, we did not investigate whether RLS treatment influenced the reported severity of RLS. Fourth, the laboratory results were collected within a week of the interview, or during the same week. Thus, it appears biased. We did not assess the influence of RLS on maternal and fetal pregnancy outcomes, which is another limitation. Lastly, the absence of a control group (women who are not pregnant) also undermines our results.

## Conclusions

The prevalence and severity of RLS during pregnancy were shown to be high, particularly in the third trimester. Age, BMI, gravida, previous RLS history, family RLS history, anemia, folate, and vitamin B12 deficiency were found to be associated with RLS during pregnancy. RLS during pregnancy requires more care to prevent or manage this syndrome.
